# Human *vs*. artificial intelligence: Physicians outperform ChatGPT in real‐world pharmacotherapy counselling

**DOI:** 10.1002/bcp.70321

**Published:** 2025-10-25

**Authors:** Benjamin Krichevsky, Stefan Engeli, Stefanie M. Bode‐Böger, Felix Koop, Martin Schulze Westhoff, Sebastian Schröder, Carsten Schumacher, Thorben Pape, Dirk O. Stichtenoth, Johannes Heck

**Affiliations:** ^1^ Hannover Medical School Institute for General Practice and Palliative Care Hannover Germany; ^2^ Interdisciplinary Emergency Department University Hospital Schleswig‐Holstein (UKSH), Campus Kiel Germany; ^3^ Institute for Pharmacology University Medicine Greifswald Greifswald Germany; ^4^ Institute for Clinical Pharmacology Otto von Guericke University of Magdeburg Magdeburg Germany; ^5^ Hannover Medical School Institute for Clinical Pharmacology Hannover Germany; ^6^ Department of Internal Medicine II, Division of Clinical Toxicology Technical University of Munich Munich Germany; ^7^ Department of Psychiatry, Social Psychiatry and Psychotherapy Hannover Medical School Hannover Germany; ^8^ PRACTIS Clinician Scientist Program, Dean's Office for Academic Career Development Hannover Medical School Hannover Germany; ^9^ Center for Clinical Trials Hannover Medical School Hannover Germany; ^10^ Department of Respiratory Medicine and Infectious Diseases Hannover Medical School Hannover Germany

**Keywords:** artificial intelligence, chatbot, ChatGPT, clinical pharmacology, drug information centre, patient safety

## Abstract

**Aims:**

To assess the utility of the artificial intelligence (AI) chatbot ChatGPT (openly available version 3.5) in responding to real‐world pharmacotherapeutic queries from healthcare professionals.

**Methods:**

Three independent and blinded evaluators with different levels of medical expertise and professional experience (*beginner*, *advanced*, and *expert*) compared AI chatbot‐ and physician‐generated responses to 70 real‐world pharmacotherapeutic queries submitted to the clinical‐pharmacological drug information centre of Hannover Medical School between June and October 2023 with regard to quality of information, answer preference, answer correctness and quality of language. Inter‐rater reliability was assessed with Krippendorff's alpha. Two separate investigators not otherwise involved in the conduct or analysis of the study selected the top three clinically relevant errors in chatbot‐ and physician‐generated responses.

**Results:**

All three evaluators rated the quality of information of physician‐generated responses higher than the quality of information of AI chatbot‐generated responses and, accordingly, thought that the physician‐generated responses were better than the chatbot‐generated responses (answer preference). All evaluators detected factually wrong information more frequently in chatbot‐generated responses than in physician‐generated responses. Although the *beginner* and *expert* evaluators rated the quality of language of physician‐generated responses higher than the quality of language of chatbot‐generated responses, there was no significant difference according to the *advanced* evaluator.

**Conclusions:**

ChatGPT's responses to real‐world pharmacotherapeutic queries were substantially inferior compared to conventional physician‐generated responses with regard to quality of information and factual correctness. Our study suggests that to date it must be strongly cautioned against the use of ChatGPT in pharmacotherapy counselling.

**Abbreviations**
ACE: angiotensin‐converting enzyme
ADR: adverse drug reaction
AI: artificial intelligence
ASA: acetylsalicylic acid
CA: California
CNS: central nervous system
COPD: chronic obstructive pulmonary disease
CRediT: Contributor Role Taxonomy
CRP: C‐reactive protein
DHPC: direct healthcare professional communication
DIC: drug information centre
DOAC: direct oral anticoagulant
ECG: electrocardiogram
eGFR: estimated glomerular filtration rate
F33.1: ICD‐10 code for recurrent depressive disorder, current episode moderate
GPT: generative pre‐trained transformer
gtt.: drops (Latin: *guttae*)
ICD‐10: International Statistical Classification of Diseases and Related Health Problems 10th Revision
INR: international normalized ratio
IQR: interquartile range
IU: international units
K‐CBMSE: Korean Comprehensive Basic Medical Sciences Examination
LLM: large language model
NSAID: non‐steroidal anti‐inflammatory drug
PDI: pharmacodynamic interaction
PKI: pharmacokinetic interaction
RIVA: Ramus interventricularis anterior
SmPC: summary of product characteristics
SSRI: selective serotonin reuptake inhibitor
TDM: therapeutic drug monitoring
USMLE: US Medical Licensing Examination

**What is known about this subject**

- Artificial intelligence (AI) chatbots such as OpenAI's ChatGPT have gained increasing popularity and their utility in medical settings has been tested across a wide spectrum of disciplines including dermatology, gynaecology, radiology and clinical pharmacy.
- Compared to other medical specialties, there is limited information on the utility of AI chatbots in clinical pharmacology.

**What this study adds**

- ChatGPT's responses to real‐world pharmacotherapeutic queries were substantially inferior to conventional physician‐generated responses with regard to quality of information and factual correctness.
- To date, the use of ChatGPT (version 3.5) in pharmacotherapy counselling must be strongly discouraged. Healthcare professionals should not (exclusively) rely on pharmacotherapeutic advice provided by AI chatbots.

## 1 INTRODUCTION

Drug information centres (DICs) significantly contribute to the dissemination of rational drug information to healthcare professionals around the globe, thus promoting the safe and effective use of drugs.^1^ DICs have a decade‐long history—the first DIC was established at the University of Kentucky in 1962^2^—and are now present in most countries worldwide.^1^ DICs are predominantly located at or affiliated with university hospitals, whereas smaller and more peripheral clinics are often not able to afford such an expert service.^3^ Hence, physicians and other healthcare professionals outside of university hospitals might be looking for alternative sources of reliable drug information and pharmacotherapeutic advice. One such possible alternative might have arisen with the advent of openly available artificial intelligence (AI) tools like ChatGPT.^4^ Developed by OpenAI (San Francisco, CA, USA) and launched to the public in November 2022, ChatGPT is based on the generative pre‐trained transformer (GPT) architecture, was trained on extensive text datasets (more than 170 billion parameters) in multiple languages and is able to generate human‐like responses to text prompts with astonishing proficiency.^5^, ^6^ ChatGPT is easily accessible, and its basic version can be used free of charge. As information on drugs continues to expand and clinical–pharmacological decision‐making becomes increasingly complex, AI tools like ChatGPT could prove useful in assisting physicians to make informed, rational and timely pharmacotherapeutic decisions.

Much has been debated about how AI tools in general will change the profession of physicians.^6^, ^7^ To date, more than 500 AI applications have been approved for use in patient care in the United States, and their number is expected to rise tremendously.^8^ AI applications have been used across a wide spectrum of medical specialties such as dermatology (e.g. identification of skin cancer^9^), gynaecology (e.g. detection of breast cancer^10^) and radiology (e.g. recognition of lung nodules^11^), with a performance similar to human experts.^8^

Jung et al demonstrated that ChatGPT (version 3.5) was able to pass the first and second German medical state examinations (if image questions were excluded) with an answer correctness of 60.1% and 66.7%, respectively,^12^ and thus achieved a similar result as in the US Medical Licensing Examination (USMLE).^13^ Of note, the best result in the second German medical state examination was achieved by ChatGPT in the field of pharmacology, with an answer correctness of 94.7%.^12^ The authors hypothesized that ChatGPT's outstanding performance on pharmacological questions might be explained by structured and publicly available information on drugs.^12^ This is supported by a study by Choi, in which ChatGPT outperformed Korean medical students in answering pharmacological multiple‐choice questions from the Korean Comprehensive Basic Medical Sciences Examination (K‐CBMSE) (overall accuracy 76% *vs*. 55%).^14^ However, ChatGPT underperformed in the subdomain of problem‐solving (answer accuracy 45%), suggesting that ‘ChatGPT is still limited in its ability to apply critical thinking and reasoning skills to real‐world problems’.^14^

To date, only sparse data are available about the utility and performance of AI tools in clinical pharmacology under real‐world conditions.^15^ Notwithstanding, Ryan et al described areas in which AI may potentially be useful in clinical pharmacology in the future, including drug discovery and development, clinical trials, medication management, personalized medicine, pharmacogenomics and pharmacovigilance.^16^ More specifically, Shahin et al suggested domains in which large language models (LLMs) such as ChatGPT might be able to aid clinical pharmacologists; these comprise editorial assistance, literature review, automation of routine tasks (e.g. translation of documents, proofreading, drafting emails, etc.), clinical trial optimization, personalized medicine and real‐world evidence analysis.^17^ ChatGPT was able to accurately describe indications, disadvantages and potential risks associated with long‐term therapy with proton pump inhibitors,^18^, ^19^ but concerns have been raised whether the drug information provided by ChatGPT is independent of the interests of the pharmaceutical industry or other stakeholders.^18^ AI‐based approaches have also been used to predict drug–drug interactions; however, the majority of current prediction models are restricted to interactions between two drugs,^20^ which represents a substantial limitation in view of prevalent polypharmacy encountered in older patients.^21^

Perhaps the most significant pitfall regarding the use of AI chatbots in medicine to date is that they tend to compose authoritative‐ and plausible‐sounding responses, which are in fact completely fabricated (including referencing non‐existent sources)—a phenomenon known as ‘hallucination’ or ‘confabulation’.^6^, ^7^, ^22^, ^23^ This has led to extreme caution regarding the potential usefulness of LLMs like ChatGPT in clinical practice.^7^, ^23^

The importance of trustworthiness and trust in the use of AI‐based systems was reviewed by Schlicker et al.^24^ The authors concluded that ‘too much trust can result in physicians uncritically relying on this technology, while too little trust may result in physicians not taking advantage of the full potential of AI‐based technology in making decisions. To strike a balance between these extremes it is crucial to correctly assess the trustworthiness of a system’.^24^

The provision of drug information and pharmacotherapeutic advice are key tasks of DICs.^3^ Inquiring healthcare professionals must be able to rely on the veracity of the provided information in their clinical decision‐making, be it a human expert or an AI chatbot that responds. The aim of our study was to evaluate the trustworthiness of ChatGPT (version 3.5) in the provision of drug information and pharmacotherapeutic advice. To this end, we compared ChatGPT's performance on 70 real‐world pharmacotherapeutic queries with the performance of physicians working at the clinical–pharmacological DIC of Hannover Medical School. The study's findings will contribute to our understanding of ChatGPT's capabilities and limitations in addressing complex clinical–pharmacological queries under real‐world conditions.

## 2 METHODS

### 2.1 Ethics approval

This study adheres to the Declaration of Helsinki (1964) and its later amendments (latest version from 2024). The Ethics Committee of Hannover Medical School approved the conduct of the study (no. 10919_BO_K_2023 and amendment).

### 2.2 Clinical–pharmacological drug information centre of Hannover Medical School

The *modus operandi* and staff composition of the clinical–pharmacological DIC of Hannover Medical School were comprehensively described by Heck et al.^3^ In brief, the DIC is a physician‐led pharmacotherapeutic consultation service that supports healthcare professionals at Hannover Medical School, a large university hospital in northern Germany, and its affiliated academic teaching hospitals and practices. Pharmacotherapeutic queries submitted to the DIC are attended to by a team of physicians and pharmacists, who draft a preliminary response. Every response draft is discussed with and authorized by a senior physician (specialist in clinical pharmacology or specialist in internal medicine) before the final response is delivered to the inquiring healthcare professional.

### 2.3 Data acquisition

At the DIC of Hannover Medical School, queries and corresponding responses are stored in anonymized form in a password‐protected Microsoft Access 2016 database (Redmond, Washington, USA).^3^ For the purpose of this study, the following parameters were retrieved for each query:

- Type of query: patient‐specific (i.e. queries referring to individual patients) or general (i.e. queries of broader pharmacotherapeutic interest or queries referring to larger patient populations, such as older patients, patients with renal insufficiency or pregnant women)
- Age and sex of the patient (only for patient‐specific queries)
- Origin of the query, for example, Hannover Medical School, academic teaching hospital, etc.
- Profession of the inquiring healthcare professional, for example, physician, dentist, etc.
- Medical specialty of the inquiring healthcare professional, for example, internal medicine, surgery, etc.
- Query categories (assignment of more than one category per query was possible): adverse drug reaction (ADR); indication/contraindication; posology/dose adjustment (e.g. due to renal insufficiency); therapeutic drug monitoring (TDM); pharmacogenetics; pharmacodynamic interaction (PDI); pharmacokinetic interaction (PKI); pregnancy and breastfeeding; pharmacotherapy in older patients (i.e. ≥65 years of age); other

Of note, the category ‘pharmacotherapy in older patients’ was not automatically assigned to queries related to patients of chronological age ≥65 years, but only if the inquiring healthcare professionals explicitly asked about drug specifics in advanced age.^3^

### 2.4 AI chatbot

ChatGPT 3.5 (versions between 24 May 2023 and 25 September 2023; OpenAI, San Francisco, USA; https://chatgpt.com) was used to generate AI chatbot responses to pharmacotherapeutic queries. Each query was entered with its original text (i.e. in German language without prior translation to English, without specification of unexplained medical abbreviations and without further instructions such as role‐specific prompts) into a fresh chatbot session. Chatbot answers were not rated in order to minimize bias due to machine learning. For the chatbot answers, no restrictions with regard to verbosity were specified.

Importantly, chatbot‐generated responses were used only for scientific purposes within the constraints of this study; they were not used for pharmacotherapeutic decisions in clinical practice.

### 2.5 Research questions and sample size calculation

The primary research question of this study was to compare the quality of information (assessed on a 5‐point Likert scale: 1 = *very poor*; 2 = *poor*; 3 = *acceptable*; 4 = *good*; 5 = *very good*; analogous to the study by Ayers et al^25^) between physician‐ and AI chatbot‐generated responses to real‐world pharmacotherapeutic queries. Quality of information was defined as a subjective measure that comprises the evaluation of the following aspects: scientific validity (the response is based on high‐quality evidence); up‐to‐dateness (the response reflects the most current knowledge available); comprehensiveness and completeness (the response addresses all critical aspects of the query and does not omit crucial information); clinical relevance, actionability and usefulness (the response aids in clinical decision‐making); parsimony (the response does not centre on irrelevant or redundant information); transparency (the response acknowledges uncertainties or limitations in the available evidence); patient‐centredness (the response takes patient‐specific factors into consideration [applicable only to patient‐specific queries]); balanced assessment of risks and benefits. Physician‐ and chatbot‐generated responses were assessed by three independent and blinded evaluators (for details, see below).

A sample size calculation was conducted with G*Power 3.1^26^, ^27^ and yielded a minimum target sample of 67 queries, assuming 80% power to detect differences between physician‐ and chatbot‐generated responses on an alpha level of .05 (Mann–Whitney *U* test) and expecting a medium effect size (Cohen's *d* = .5; the estimated effect size was based on previous studies^25^, ^28^). Hence, 70 consecutive queries submitted to the clinical–pharmacological DIC of Hannover Medical School between June and October 2023 were included in the study.

Explorative research questions comprised the evaluators' preferences for either physician‐ or chatbot‐generated responses (‘Which response do you think is better?’), the correctness of the responses (‘Did you detect any factually wrong information in the response?’) and the quality of language of the responses (assessed on a 5‐point Likert scale from 1 = *very poor* to 5 = *very good*). Quality of language was defined as a subjective measure that encompasses the evaluation of the following aspects: clarity, structure, readability and comprehensibility of the text (the response is clearly written, well‐organized and easily readable and understandable); grammar, syntax, spelling and punctuation; appropriate and consistent use of medical and clinical–pharmacological terminology; professional tone and fluency; and contextual sensitivity (the response takes the inquirer's professional background into consideration). The evaluators had the possibility to comment their answers.

In addition, the answer length (word count) was compared between physician‐ and chatbot‐generated responses. Finally, the proportions of responses that the evaluators were able to correctly identify as created by AI were assessed to determine whether there was evidence of (unintended but de facto) evaluator unblinding.

### 2.6 Evaluators and evaluator blinding

Three evaluators (FK, BK and SE) with different levels of medical expertise and professional experience independently assessed the physician‐ and AI chatbot‐generated responses. FK is a resident in internal medicine and clinical toxicology (1 year of professional experience; hereon referred to as *beginner*). BK is a resident in internal medicine (4 years of professional experience; hereon referred to as *advanced*). SE is a specialist in clinical pharmacology (23 years of professional experience; hereon referred to as *expert*).

Similar to the study by Ayers et al,^25^ any revealing information was removed from the responses (e.g. sentences such as ‘I am an artificial intelligence’). Subsequently, responses were randomly ordered and labelled response A or response B to blind evaluators. The evaluators were then shown the entire query, the physician‐generated response and the chatbot‐generated response (in blinded fashion).

### 2.7 Statistical analysis

Descriptive statistical techniques were used to summarize the data. Quantitative variables were tested for normal distribution with the Shapiro–Wilk test and by inspection of histograms and Q–Q plots. Since all quantitative variables were not normally distributed, medians with interquartile ranges (IQRs) were reported (instead of means with standard deviations). For categorical variables, absolute and relative frequencies are shown. Differences between physician‐ and AI chatbot‐generated responses were analysed with Mann–Whitney *U* test or Pearson's chi‐squared test, as appropriate. *P*‐values <.05 were considered statistically significant. Due to the exploratory nature of our study, no adjustments for multiple testing were made.

To assess inter‐rater reliability between the three evaluators, Krippendorff's alpha with 95% confidence intervals (CIs) was calculated (number of bootstrap samples: 10 000).^29^ Inter‐rater reliabilities were interpreted as follows: Krippendorff's alpha ≥.800: *reliable*; .800 > Krippendorff's alpha ≥.667: *moderate*; Krippendorff's alpha <.667: *insufficient*.^30^

All statistical analyses were conducted with IBM SPSS Statistics for Windows, version 29 (Armonk, New York, USA). For the calculation of Krippendorff's alpha, the KAPLHA macro for SPSS by Hayes and Krippendorff was applied.^29^

## 3 RESULTS

### 3.1 Number, type and origin of queries

Seventy consecutive queries submitted to the clinical–pharmacological DIC of Hannover Medical School between June and October 2023 were analysed. Of these, 97.1% (68/70) were patient‐specific queries, while the remaining 2.9% (2/70) were general queries (Table 1). The majority of queries were submitted by physicians (97.1%; 68/70), and 91.4% (64/70) came from healthcare professionals employed at Hannover Medical School. Surgeons, internists and psychiatrists most frequently consulted the DIC, submitting 27.1% (19/70), 20.0% (14/70) and 17.1% (12/70) of all queries, respectively.

**TABLE 1:** Characteristics of 70 consecutive queries submitted to the clinical‐pharmacological drug information centre of Hannover Medical School between June and October 2023.

Variables	Number of submitted queries	%
Type of query		
Patient‐specific	68	97.1
General	2	2.9
Origin of query		
Hannover Medical School	64	91.4
Academic teaching practice	3	4.3
Academic teaching hospital	2	2.9
Alumni of Hannover Medical School	1	1.4
Profession of inquiring healthcare professional		
Physician	68	97.1
Medical student	2	2.9
Specialty of inquiring healthcare professional		
Surgery	19	27.1
Internal medicine	14	20.0
Psychiatry and psychosomatic medicine	12	17.1
Urology	8	11.4
Gynaecology and obstetrics	5	7.1
Paediatrics	3	4.3
Radiology and radiotherapy	3	4.3
Neurology	2	2.9
Ophthalmology	1	1.4
Otorhinolaryngology	1	1.4
Human genetics	1	1.4
Not documented	1	1.4
Query category (assignment of more than one category per query was possible)		
Pharmacodynamic interaction	37	52.9
Indication/contraindication	34	48.6
Adverse drug reaction	23	32.9
Pharmacokinetic interaction	23	32.9
Posology/dose adjustment (e.g. due to renal or hepatic insufficiency)	21	30.0
Pharmacotherapy in older patients	7	10.0
Therapeutic drug monitoring	7	10.0
Pharmacogenetics	2	2.9
Pregnancy and breastfeeding	1	1.4
Other	2	2.9

### 3.2 Patient characteristics

Age and sex of patients were documented in 86.8% (59/68) and 98.5% (67/68) of patient‐specific queries, respectively. The median age of patients in patient‐specific queries was 62 years (IQR 44–79 years, range 1–99 years), and 51.5% (35/68) of the patients were female (male: 47.1% (32/68); sex not documented: 1.5% [1/68]) (Table 1).

### 3.3 Query categories

Pharmacodynamic interactions were involved in more than half of all queries (52.9%; 37/70), followed by indications/contraindications (48.6%; 34/70), adverse drug reactions and pharmacokinetic interactions (each 32.9%; 23/70) (Table 1).

### 3.4 Primary research question

All three evaluators rated the quality of information of physician‐generated responses higher than the quality of information of AI chatbot‐generated responses (median score [IQR] physician‐generated responses *vs*. chatbot‐generated responses: *beginner*, 5 [5–5] *vs*. 2 (2–3), *p* < .001; *advanced*, 5 [4–5] *vs*. 2 [2–3], *p* < .001; *expert*, 5 [4–5] *vs*. 2 [1–2], *p* < .001; Table 2). This corresponded to a *very good* quality of information for physician‐generated responses compared to a *poor* quality of information for chatbot‐generated responses. The inter‐rater reliability for the evaluation of the quality of information was *moderate* (Krippendorff's alpha = .789; 95% CI 0.759–0.817).

**TABLE 2:** Reviewers' assessments for the primary and explorative research questions.

Research questions	Beginner	*p* value (beginner)	Advanced	*p* value (advanced)	Expert	*p* value (expert)
Primary research question						
Quality of information						
Median score physician‐generated responses (IQR)	5 (5–5)	<.001^a^	5 (4–5)	<.001^a^	5 (4–5)	<.001^a^
Median score AI chatbot‐generated responses (IQR)	2 (2–3)		2 (2–3)		2 (1–2)	
Explorative research questions						
Which response do you think is better?						
Physician‐generated response; *n* (%)	69 (98.6)	<.001^b^	68 (97.1)	<.001^b^	70 (100)	<.001^b^
AI chatbot‐generated response; *n* (%)	1 (1.4)		2 (2.9)		0 (0)	
Did you detect any factually wrong information in the response?						
Physician‐generated response; *n* (%)	4 (5.7)	<.001^b^	5 (7.1)	<0.001^b^	12 (17.1)	<.001^b^
AI chatbot‐generated response; *n* (%)	32 (45.7)		23 (32.9)		39 (55.7)	
Quality of language						
Median score physician‐generated responses (IQR)	5 (5–5)	<0.001^a^	5 (4–5)	0.559^a^	4 (4–4)	<.001^a^
Median score AI chatbot‐generated responses (IQR)	4 (4–5)		5 (4–5)		3 (3–3)	

*Note*: Assessments were conducted by three independent and blinded evaluators with different levels of medical expertise and professional experience (beginner, advanced and expert). Quality of information and quality of language of physician‐ and AI chatbot‐generated responses were assessed on a 5‐point Likert scale from 1 = *very poor* to 5 = *very good*. Reviewers' preferences of responses (‘Which response do you think is better?’) and correctness of responses (‘Did you detect any factually wrong information in the response?’) were assessed in binary form (answer possibilities: ‘physician‐generated response’ *vs*. ‘AI chatbot‐generated response’, and ‘yes’ *vs*. ‘no’, respectively). Significant *p* values are highlighted in bold.

Abbreviations: AI, artificial intelligence; IQR, interquartile range.

^a^ Mann–Whitney *U* test.

^b^ Pearson's chi‐squared test.

### 3.5 Explorative research questions

In addition, the *beginner*, *advanced* and *expert* evaluators thought that the physician‐generated responses were better than the AI chatbot‐generated responses (binary assessment) in 98.6% (69/70), 97.1% (68/70) and 100% (70/70) of cases, respectively (all *p* < .001; Table 2). The inter‐rater reliability for the evaluator preference was *reliable* (Krippendorff's alpha = .943; 95% CI 0.910–0.972).

All evaluators detected factually wrong information more frequently in chatbot‐generated responses than in physician‐generated responses (proportion of responses containing factually wrong information [according to evaluators] of physician‐generated responses *vs*. chatbot‐generated responses: *beginner*, 5.7% [4/70] *vs*. 45.7% [32/70], *p* < .001; *advanced*, 7.1% [5/70] *vs*. 32.9% (23/70), *p* < .001; *expert*, 17.1% [12/70] *vs*. 55.7% [39/70], *p* < .001; Table 2). The inter‐rater reliability for the detection of factually wrong information was *insufficient* (Krippendorff's alpha = .486; 95% CI 0.391–0.582).

The *beginner* and *expert* evaluators rated the quality of language of physician‐generated responses higher than the quality of language of chatbot‐generated responses, whereas there was no difference according to the *advanced* evaluator (median score [IQR] physician‐generated responses *vs*. chatbot‐generated responses: *beginner*, 5 [5–5] *vs*. 4 [4–5], *p* < .001; *advanced*, 5 [4–5] *vs*. 5 [4–5], *p* = .559; *expert*, 4 [4–4] *vs*. 3 [3–3], *p* < .001; Table 2). The inter‐rater reliability for the evaluation of the quality of language was *insufficient* (Krippendorff's alpha = −.160; 95% CI –.268 to −.052).

### 3.6 Answer length

Physician‐ and AI chatbot‐generated responses had a median word count of 77 words (IQR 44–125; range 13–378) and 186 words (IQR 122–253; range 46–441), respectively (*p* < .001).

### 3.7 Identification of responses as created by AI

The *beginner*, *advanced* and *expert* evaluators correctly identified chatbot‐generated responses as created by AI in 98.6% (69/70), 100% (70/70) and 100% (70/70) of cases.

### 3.8 Errors in physician‐ and chatbot‐generated responses

To err is human but also AI chatbots are known to occasionally produce factually wrong information that sounds plausible and authoritative, a phenomenon termed ‘hallucination’ or ‘confabulation’.^6^, ^7^ Hence, two investigators not otherwise involved in the conduct and analysis of the study (MSW and SS) screened the physician‐ (MSW) and chatbot‐generated responses (SS) for errors. Their subjective selections of and comments on the three most clinically relevant errors in physician‐ and chatbot‐generated responses are showcased in Data S1. For the selected cases, the corresponding chatbot‐ or physician‐generated responses (respectively) are juxtaposed in the last column for comparison.

## 4 DISCUSSION

In this study, three independent and blinded evaluators with different levels of medical expertise and professional experience unanimously rated the quality of information of AI chatbot (ChatGPT 3.5)‐generated responses to real‐world pharmacotherapeutic queries as *poor*, compared to a *very good* quality of information of conventional physician‐generated responses. In accordance, the evaluators preferred the physician‐generated responses to the chatbot‐generated responses in 97%–100% of cases. The poor results of ChatGPT in our study with respect to quality of information and factual correctness may partly be explained by the circumstance that queries were entered in German, whereas ChatGPT was predominantly (93%) trained on datasets in English language^31^ and without medical focus.^7^, ^12^, ^31^ Besides German, the problem of inferior ChatGPT answer quality compared to English has also been noticed for other languages, such as French or Arabic.^32^

The quality of language of physician‐generated responses was also rated higher than the quality of language of chatbot‐generated responses in our study, but the differences were smaller and not as consistent across the evaluators as for quality of information. This result supports the idea that AI chatbots are first and foremost tools for text creation and not primarily a source of reliable medical information.^4^

The inter‐rater reliability (assessed with Krippendorff's alpha) was heterogeneous with respect to the different research questions, ranging from *insufficient* to *reliable*. Therefore, only tentative conclusions should be drawn from our study results.^30^ It should be noted, however, that Krippendorff's alpha is considered a very conservative measure for inter‐rater reliability.^33^

Query characteristics in the present study were overall comparable to our previous evaluation of the clinical‐pharmacological DIC of Hannover Medical School.^3^ While the proportion of patient‐specific queries was higher in the present study (97.1% *vs*. 82.8%^3^), the proportion of inquiring healthcare professionals who were physicians was very similar (97.1% *vs*. 96.1%^3^). Also, the specialties of the inquiring healthcare professionals who most frequently consulted the DIC (i.e. internal medicine, surgery and psychiatry and psychosomatic medicine) as well as the addressed query categories (i.e. ADRs, indications/contraindications, PDIs and PKIs) were identical in both studies.^3^ Furthermore, age and sex distributions of patient‐specific queries were comparable (median age 62 years [IQR 44–79] *vs*. median 60 years [IQR 37–72],^3^ and 51.5% *vs*. 51.4% female patients,^3^ respectively).

The rates of factually wrong information detected in physician‐generated responses in this study (5.7%–17.1%) may appear relatively high; however, it could be retrieved from the evaluators' comments that the evaluators also partly considered omitted information as errors, which may explain the relatively high error rate of physicians. As expected, the detection rates of factually wrong information in physician‐ and also AI chatbot‐generated responses reflected the evaluators' expertise, with the *expert* detecting more errors than the *beginner* and the *advanced*.

Two investigators not otherwise involved in the conduct and analysis of this study retrieved the three most clinically relevant errors in physician‐ and AI chatbot‐generated responses. According to their subjective assessments, the three most clinically relevant errors made by physicians were: (1) not addressing the increased risk of bleeding under treatment with acetylsalicylic acid and tinzaparin; (2) not mentioning moxonidine as a potential cause of depression; and (3) not considering the potential interaction between ramipril and potassium with the increased risk of hyperkalaemia. As described above, those errors by physicians can rather be characterized as omissions of clinically relevant information than as errors in the strict sense. Intensive monitoring strategies especially with regard to potential bleeding signs and measurement of electrolyte serum levels should have additionally been recommended to avert clinical consequences.

On the other hand, the three most clinically relevant errors made by ChatGPT were (1) the description of tazobactam as a sedative agent, suggesting a potential interaction with hydromorphone; (2) the description of Actrapid as an analgesic; and (3) recommending INR monitoring for direct oral anticoagulants. Hypothetically, if healthcare professionals had followed the chatbot's advice in the real world, false pharmacotherapeutic conclusions might have been drawn, such as potentially avoiding the (typically unproblematic) combination of tazobactam and hydromorphone due to (unfounded) fear of excessive sedation, insufficient pain management and/or risk of hypoglycaemia if Actrapid had been used as an analgesic (which it is not) and risk of bleeding or risk of thromboembolic events if the INR had been used for therapy monitoring of direct oral anticoagulants (for whom it is not a suitable monitoring parameter, in contrast to vitamin K antagonists).

Taken together, errors made by ChatGPT were generally much more serious than errors made by physicians and putatively would have had much more far‐reaching clinical consequences if they had been implemented in the real world. Of note, errors by AI chatbots are not only common to clinical pharmacology but have also been reported in cardiology where, for example, ChatGPT falsely assigned the RIVA (Ramus interventricularis anterior) to the right coronary artery.^34^ In conclusion, it is currently indispensable to thoroughly check AI chatbot‐generated output for quality of information and factual correctness.^34^

Ayers et al found that ChatGPT was able to generate responses to patient questions posed in an online social media forum that were rated by three evaluators to be of higher quality and empathy than physician‐generated responses.^25^ In our study, by contrast, the quality of information of ChatGPT‐generated responses was markedly lower than the quality of information of physician‐generated responses. However, the queries in our study were submitted by healthcare professionals, mostly physicians, and addressed complex pharmacotherapeutic topics. This suggests that while ChatGPT may be able to adequately respond to lay questions, it may struggle with competing at a healthcare professional level of communication. Ayers et al suggested that chatbots might be used in clinical settings ‘to draft responses that physicians could then edit’.^25^ However, the evaluators in our study detected that the proportion of responses that contained factually wrong information was 3–8 times higher for chatbot‐generated responses than for physician‐generated responses. Hence, at the current stage, physicians would not only have to edit chatbot‐drafted responses but they would have to actively search for potential errors and rectify them, a task that in some instances might be even more tedious and time‐consuming than elaborating responses themselves. On the other hand, there might also be occasions where editing a chatbot‐drafted response is faster and more efficient than creating a response on one's own from scratch. In our study, the time required by human experts *vs*. ChatGPT to complete their assessments was not formally measured. Experience suggests that while human experts typically needed approximately 30–120 min to complete their responses, ChatGPT only required a couple of seconds to compile its answers. Future studies should systematically investigate the time needed to finalize responses (drafting plus editing) of physician‐ *vs*. chatbot‐generated answers to capture and quantify potential differences rigorously.

Interestingly, answer lengths were similar between our investigation and the Ayers et al study (physician‐generated responses: median 77 [IQR 44–125] *vs*. 52 [17–62] words^25^; chatbot‐generated responses: median 186 [IQR 122–253] *vs*. 211 [168–245] words^25^), with significantly longer chatbot‐generated responses compared to physician‐generated responses in both studies. With the 5‐point Likert scale we also adopted a similar methodology as Ayers et al,^25^ which increases comparability. An advantage of our investigation is that the evaluators assessed physician‐ and chatbot‐generated responses for factual accuracy, which was not the case in the Ayers et al study.^25^

Goodman et al evaluated ChatGPT‐generated answers to physician‐generated medical queries (*n* = 284) and found a median accuracy score of 5.5 (IQR 4–6) on a Likert scale from 1 = *completely incorrect* to 6 = *completely correct*, suggesting an overall excellent chatbot performance in their study.^35^ However, their set of queries only contained ‘questions with clear and uncontroversial answers from available medical guidelines’^35^ and not real‐world cases as in our investigation. Besides, the Goodman et al study was conducted without a human control group and in an unblinded fashion, limiting its internal and external validity.^35^

To date, information on the utility of ChatGPT in clinical pharmacology is limited.^15^, ^36^, ^37^ Montastruc et al compared the quality of ChatGPT‐ and human specialist‐generated responses to queries sent to the Toulouse Pharmacovigilance Center.^15^ Their study sample was smaller compared to our investigation (50 *vs*. 70 queries), was not based on a formal sample size calculation, was not blinded, contained both oral and written queries and included queries from healthcare professionals as well as from patients.^15^ Our study sample, by contrast, was conducted in a blinded fashion (although there was evidence of de facto evaluator unblinding), featured a formal sample size calculation and exclusively consisted of written queries by healthcare professionals. Montastruc et al used ChatGPT version 4.0,^15^ whereas our study was conducted with the older but openly accessible version 3.5. Despite these differences in study set‐up, however, the conclusions that can be drawn from the Montastruc et al study^15^ and from our investigation appear similar: The quality of ChatGPT‐generated responses is currently insufficient for routine use in clinical pharmacology.

In a study by Helgestad et al, by contrast, ChatGPT 3.5 and 4.0 provided responses to medication‐related queries submitted by clinicians to the clinical–pharmacological counselling service of the Central and North Denmark regions, which were rated better than or equal to physician‐generated answers in 80% (39/49) and 98% (48/49) of cases, respectively.^37^ However, the evaluators in that study were neither independent nor blinded, nor was a sample size calculation conducted, limiting the validity of the results.^37^

Besides clinical pharmacology, several studies investigated ChatGPT's performance in clinical pharmacy and showed a highly variable accuracy rate of 26%–100% in answering medication‐related questions.^23^, ^28^, ^38^, ^39^, ^40^, ^41^, ^42^, ^43^ Huang et al tested the capabilities and limitations of ChatGPT in clinical pharmacy and found that it performed significantly weaker than humans in the domains prescription review, ADR recognition, ADR causality assessment and patient medication education. Only in drug counselling was ChatGPT's performance similar (but not superior) to a human clinical pharmacist.^38^ The authors concluded that ChatGPT ‘lacked (…) the ability for handling advanced reasoning and complex instructions’,^38^ a notion that we can adopt for clinical pharmacology based on the results of our study. One of ChatGPT's limitations described by Huang et al was ‘insufficient integration of patients' real‐life circumstances’.^38^ As 97% of the queries analysed in our study were patient specific, this may explain ChatGPT's weak performance in our investigation.

It should be mentioned that ChatGPT (version 4) surpassed clinical pharmacists in another study (79% *vs*. 66% accurate responses).^44^ ChatGPT's domains of excellence were oncology, nephrology and psychiatry.^44^ The questions in that study, however, were multiple‐choice questions with four answering options from which ChatGPT had to choose the correct one. Hence, the study setting differed markedly from our investigation in which ChatGPT was asked to formulate free‐text answers to clinically complex open‐ended pharmacotherapeutic queries.

Morath et al investigated the performance and risks associated with the usage of ChatGPT to answer drug‐related questions.^28^ Similar to Montastruc et al, Morath et al only tested 50 queries without formal sample size calculation. On the other hand, there were six evaluators in the Morath et al study (compared to three evaluators in our investigation), and—in contrast to our study—reproducibility of chatbot responses was also analysed. Lack of answer reproducibility has been described as a major concern in the use of AI chatbots in medical practice.^45^ Interestingly, the proportion of false chatbot answers in the Morath et al study was in the range of our study (38.0%^28^ and 32.9%–55.7%, respectively). Also similar to the Morath et al. study, no references were provided by ChatGPT in our investigation, seriously limiting traceability and transparency of chatbot responses. This finding may inform future studies in which AI chatbots that are in principle able to reference their responses should be evaluated, such as Perplexity (San Francisco, CA, USA; https://www.perplexity.ai/). In contrast to Morath et al,^28^ we did not translate the queries from German to English before entering them into ChatGPT in order to more accurately depict real‐world conditions in a German hospital. For pharmaceutical queries, Morath et al concluded that ‘the use of artificial intelligence applications in drug information is not possible as long as barriers like wrong content, missing references and reproducibility remain’, a notion we fully agree with for clinical pharmacology.

Haftenberger and Dierks comprehensively described the legal framework in Germany for the use of AI‐based systems by physicians.^46^ They concluded that physicians are free to choose the therapy they consider most appropriate for their patients and can thus use AI‐based systems at their discretion and judgement.^46^ Physicians must, however, be aware of AI‐specific risks and include them in their decision‐making process, be alert to anomalies and be able to respond accordingly.^46^ Physicians always remain in control of the treatment even when using AI as support and must scrutinize results obtained from AI‐based systems all the more critically the more far‐reaching the consequences of a wrong decision are.^46^ It must, however, be pointed out that the same considerations apply if physicians consult human experts instead of AI tools, for example, when submitting a pharmacotherapeutic query to a DIC. Here also, the treating physician must not uncritically adopt the answer from the DIC but still remains responsible for his or her final pharmacotherapeutic decision.

The present study has several limitations. First of all, we did not systematically assess the potential harm that would have occurred if actions had been initiated based on the erroneous information and wrong treatment recommendations provided by ChatGPT. This aspect should be addressed in future studies in clinical pharmacology because Morath et al found a high risk of severe patient harm in 26% of chatbot responses^28^ in clinical pharmacy. Future investigations should aim to quantify error severity and potential harm to patients using standardized frameworks.

Even though the evaluators in our study were formally blinded, they were able to reliably predict which answers were created by AI, resulting in a de facto unblinding and potentially leading to confirmation bias. This may be due to the evaluators' own previous experiences with publicly available AI applications. Also, the study by Ayers et al found that chatbot‐generated responses were significantly longer than physician‐generated responses,^25^ a finding that was possibly known to the evaluators. Of note, a recent study by Weis et al pointed towards a bias against medical advice when labelled as AI‐generated (‘algorithm aversion’),^47^ which might have influenced the evaluators' assessments in the present investigation. The consistently high ability of all three evaluators to distinguish between responses created by ChatGPT and those written by physicians in a binary‐choice setting indicates that notable differences remain between chatbot‐ and human expert‐generated content. Although we did not systematically examine the specific features that led evaluators to classify responses as either AI‐ or human‐generated, future research should investigate which factors—for example, response length, clinical depth and actionability or linguistic style—most strongly influence these perceptions.

The evaluators of our study are also authors of this publication, which might have influenced their independence and biased their assessments, a limitation that is similar to the Ayers et al study.^25^ Independence is a critical factor in ensuring the credibility of evaluations. However, dual roles are not inherently problematic, provided they are managed with transparency and methodological rigour. In our case, we are convinced that the engagement of evaluators as authors was both necessary and appropriate. The evaluation process required distinct levels of medical expertise and professional experience that could only be provided by investigators directly involved in the conduct of the study. Excluding these experts from authorship would have been misleading, as they made substantial intellectual contributions and assumed full responsibility for the integrity of the work. To ensure transparency and accountability, we clearly declared the dual role of these individuals as both evaluators and authors.

It might be considered a limitation that GPT‐3.5 was used in this study instead of the more advanced GPT‐4 model. However, during the time of study conduct, GPT‐3.5 was openly available to the public and therefore in more widespread use than GPT‐4, which was only available on a subscription basis.^38^ Of note, in August 2025, GPT‐5 was released,^48^, ^49^ and future studies should explore how the latest model compares to previous versions. In addition, the capabilities of other increasingly popular and widely used LLMs such as Google's Gemini^50^ or Meta's Llama^51^ in real‐world pharmacotherapy counselling should be investigated.

Finally, different results (potentially more favourable from the chatbot's point of view) might have been obtained if the queries had been translated from German to English prior to entering them into the chatbot sessions or if role‐specific prompts had been used (e.g., ‘You are an expert clinical pharmacologist. You have received the following query. Please answer as if advising on a real patient case’) since this might have affected the accuracy, depth and length of AI responses. In this context, it should also be mentioned that some queries (e.g. the second example in Data S1) lacked a clearly formulated question, which—while reflecting real‐world consultation requests—may create difficulties for AI systems that require explicit instructions. In the absence of a direct prompt or question, AI models may default to less precise or debutant‐level responses. Future investigations on the utility of AI chatbots in pharmacotherapy counselling should take these aspects into consideration when devising the study design.

The present study focused exclusively on queries from healthcare professionals and corresponding expert evaluations. Future work should also address how patients perceive AI‐generated responses to drug‐related questions, especially when the information is factually correct but varies in tone, empathy or clarity.

The use of AI assistance has already become accepted medical practice in the interpretation of radiographs, computed tomographic and magnetic resonance imaging scans, electrocardiograms, skin images and retinal photographs.^52^ In clinical pharmacology, however, AI tools have not yet demonstrated the same convincing level of performance. To date, clinical–pharmacological DICs should still adhere to their established *modi operandi* in providing pharmacotherapeutic advice; they should not rely on AI chatbots. Nonetheless, AI chatbots like ChatGPT may be used as an additional source of information besides others (i.e. drug information databases, drug compendia, textbooks, primary literature, etc.) to collect further suggestions, but all information provided by chatbots must be carefully checked for factual correctness. Yet, sophisticated knowledge is required to identify factually wrong information in chatbot responses.^28^ Teaching clinical–pharmacological skills to medical students and young physicians still remains paramount.

According to Krumborg et al, AI has great potential as a tool in clinical pharmacology, for example, as a writing aid or as a data analysis assistant in pharmacogenomics.^53^ ‘It should not, though, act as a substitute for the extensive literature review, wealth of cross‐references and sceptical mind of a clinical pharmacologist’.^53^ We fully support this conclusion. In the future, clinical pharmacologists should adopt a leading position in the curious but critical assessment of AI tools for the provision of drug information and pharmacotherapeutic advice.

## AUTHOR CONTRIBUTIONS

**Benjamin Krichevsky:** Conceptualization; formal analysis; investigation; methodology; visualization; writing—original draft. **Johannes Heck:** Conceptualization; formal analysis; methodology; project administration; visualization; writing—original draft. **Martin Schulze Westhoff:** Data curation; validation; writing—review and editing. **Sebastian Schröder:** Data curation; validation; writing—review and editing. **Carsten Schumacher:** Formal analysis; validation; writing—review and editing. **Thorben Pape:** Formal analysis; validation; writing—review and editing. **Stefan Engeli:** Investigation; writing—review and editing. **Felix Koop:** Investigation; writing—review and editing. **Stefanie M. Bode‐Böger:** Supervision; writing—review and editing. **Dirk O. Stichtenoth:** Supervision; writing—review and editing.

## CONFLICT OF INTEREST STATEMENT

The authors state that they have no conflicts of interest to declare.

## Supporting information


**Data S1.** Examples of errors in physician‐ and artificial intelligence chatbot‐generated responses to pharmacotherapeutic queries submitted by healthcare professionals to the clinical‐pharmacological drug information center of Hannover Medical School. Note: Queries and responses were translated from German to English with DeepL (Cologne, Germany; https://www.deepl.com/de/translator). For the selected cases, the corresponding chatbot‐ or physician‐generated responses (respectively) are juxtaposed in the last column for comparison. Please note that those responses may also contain errors.

## Data Availability

The data that support the findings of this study are available upon reasonable request from the corresponding author.

## References

[1] Nova Manosalva MA , López Gutiérrez JJ , Cañas M . Drug information centers: an overview to the concept. Rev Colomb Cienc Quim Farm. 2016;45(2):243‐255. doi:10.15446/rcciquifa.v45n2.59940

[2] Gabay MP . The evolution of drug information centers and specialists. Hosp Pharm. 2017;52(7):452‐453. doi:10.1177/0018578717724235 29276270 PMC5735713

[3] Heck J , Stichtenoth DO , Sabau R , et al. Clinical‐pharmacological drug information center of Hannover Medical School: experiences and analysis from a tertiary care university hospital. Sci Rep. 2022;12(1):19409. doi:10.1038/s41598-022-24005-y 36371467 PMC9653451

[4] OpenAI . Introducing ChatGPT. 2022. Available at: https://openai.com/index/chatgpt/. Accessed May 26, 2025.

[5] Ouyang L , Wu J , Jiang X , et al. Training language models to follow instructions with human feedback. NeurIPS. 2022;35:27730‐27744.

[6] Sallam M . ChatGPT utility in healthcare education, research, and practice: systematic review on the promising perspectives and valid concerns. Health. 2023;11(6):887. doi:10.3390/healthcare11060887 PMC10048148 36981544

[7] Lee P , Bubeck S , Petro J . Benefits, limits, and risks of GPT‐4 as an AI chatbot for medicine. N Engl J Med. 2023;388(13):1233‐1239. doi:10.1056/NEJMsr2214184 36988602

[8] Wehkamp K , Krawczak M , Schreiber S . The quality and utility of artificial intelligence in patient care. Dtsch Arztebl Int. 2023;120(27–28):463‐469. doi:10.3238/arztebl.m2023.0124 37218054 PMC10487679

[9] Haenssle HA , Fink C , Toberer F , et al. Man against machine reloaded: performance of a market‐approved convolutional neural network in classifying a broad spectrum of skin lesions in comparison with 96 dermatologists working under less artificial conditions. Ann Oncol. 2020;31(1):137‐143. doi:10.1016/j.annonc.2019.10.013 31912788

[10] Romero‐Martín S , Elías‐Cabot E , Raya‐Povedano JL , Gubern‐Mérida A , Rodríguez‐Ruiz A , Álvarez‐Benito M . Stand‐alone use of artificial intelligence for digital mammography and digital breast tomosynthesis screening: a retrospective evaluation. Radiology. 2022;302(3):535‐542. doi:10.1148/radiol.211590 34904872

[11] Chamberlin J , Kocher MR , Waltz J , et al. Automated detection of lung nodules and coronary artery calcium using artificial intelligence on low‐dose CT scans for lung cancer screening: accuracy and prognostic value. BMC Med. 2021;19(1):55. doi:10.1186/s12916-021-01928-3 33658025 PMC7931546

[12] Jung LB , Gudera JA , Wiegand TLT , Allmendinger S , Dimitriadis K , Koerte IK . ChatGPT passes German state examination in medicine with picture questions omitted. Dtsch Arztebl Int. 2023;120(21):373‐374. doi:10.3238/arztebl.m2023.0113 37530052 PMC10413971

[13] Kung TH , Cheatham M , Medenilla A , et al. Performance of ChatGPT on USMLE: potential for AI‐assisted medical education using large language models. PLOS Digit Health. 2023;2(2):e0000198. doi:10.1371/journal.pdig.0000198 36812645 PMC9931230

[14] Choi W . Assessment of the capacity of ChatGPT as a self‐learning tool in medical pharmacology: a study using MCQs. BMC Med Educ. 2023;23(1):864. doi:10.1186/s12909-023-04832-x 37957666 PMC10644619

[15] Montastruc F , Storck W , de Canecaude C , et al. Will artificial intelligence chatbots replace clinical pharmacologists? An exploratory study in clinical practice. Eur J Clin Pharmacol. 2023;79(10):1375‐1384. doi:10.1007/s00228-023-03547-8 37566133

[16] Ryan DK , Maclean RH , Balston A , Scourfield A , Shah AD , Ross J . Artificial intelligence and machine learning for clinical pharmacology. Br J Clin Pharmacol. 2024;90(3):629‐639. doi:10.1111/bcp.15930 37845024

[17] Shahin MH , Barth A , Podichetty JT , et al. Artificial intelligence: from buzzword to useful tool in clinical pharmacology. Clin Pharmacol Ther. 2024;115(4):698‐709. doi:10.1002/cpt.3083 37881133

[18] ChatGPT zum thema PPI‐langzeittherapie: Ist künstliche intelligenz eine quelle für unabhängige informationen zu arzneimitteln? Der Arzneimittelbrief. 2023;57(7):49‐51.

[19] Arzneimittelbrief . Long‐term PPI therapy: assessment. 2023. Available at: https://chat.openai.com/share/51012c1a-8ca9-4a4d-a0bb-f731a39c4860. Accessed May 28, 2024.

[20] Han K , Cao P , Wang Y , et al. A review of approaches for predicting drug‐drug interactions based on machine learning. Front Pharmacol. 2022;12:814858. doi:10.3389/fphar.2021.814858 35153767 PMC8835726

[21] Mosshammer D , Haumann H , Morike K , Joos S . Polypharmacy—an upward trend with unpredictable effects. Dtsch Arztebl Int. 2016;113(38):627‐633. doi:10.3238/arztebl.2016.0627 27743469 PMC5078862

[22] Li R , Kumar A , Chen JH . How chatbots and large language model artificial intelligence systems will reshape modern medicine: fountain of creativity or Pandora's box? JAMA Intern Med. 2023;183(6):596‐597. doi:10.1001/jamainternmed.2023.1835 37115531 PMC12049698

[23] Grossman S , Zerilli T , Nathan JP . Appropriateness of ChatGPT as a resource for medication‐related questions. Br J Clin Pharmacol. 2024;90(10):2691‐2695. doi:10.1111/bcp.16212 39096130

[24] Schlicker N , Langer M , Hirsch MC . How trustworthy is artificial intelligence?: a model for the conflict between objectivity and subjectivity. Inn Med (Heidelb). 2023;64(11):1051‐1057. doi:10.1007/s00108-023-01602-1 37737496

[25] Ayers JW , Poliak A , Dredze M , et al. Comparing physician and artificial intelligence chatbot responses to patient questions posted to a public social media forum. JAMA Intern Med. 2023;183(6):589‐596. doi:10.1001/jamainternmed.2023.1838 37115527 PMC10148230

[26] Faul F , Erdfelder E , Lang A , Buchner A . G*Power 3: a flexible statistical power analysis program for the social, behavioral, and biomedical sciences. Behav Res Methods. 2007;39(2):175‐191. doi:10.3758/bf03193146 17695343

[27] Faul F , Erdfelder E , Buchner A , Lang A . Statistical power analyses using G*Power 3.1: tests for correlation and regression analyses. Behav Res Methods. 2009;41(4):1149‐1160. doi:10.3758/BRM.41.4.1149 19897823

[28] Morath B , Chiriac U , Jaszkowski E , et al. Performance and risks of ChatGPT used in drug information: an exploratory real‐world analysis. Eur J Hosp Pharm. 2023;31(6):491‐497. 10.1136/ejhpharm-2023-003750 37263772

[29] Hayes AF , Krippendorff K . Answering the call for a standard reliability measure for coding data. Commun Methods Meas. 2007;1(1):77‐89. doi:10.1080/19312450709336664

[30] Krippendorff K . Content Analysis: An Introduction to Its Methodology. 2nd ed. Sage Publications; 2004.

[31] Brown T , Mann B , Ryder N , et al. Language models are few‐shot learners. NeurIPS. 2020;33:1877‐1901.

[32] Seghier ML . ChatGPT: not all languages are equal. Nature. 2023;615(7951):216. doi:10.1038/d41586-023-00680-3 36882613

[33] Dobbrunz S , Brunner F , Müller JL , Briken P . Interrater‐reliabilität der kriteriengeleiteten beurteilung der schuldfähigkeit bei paraphilen störungen. Nervenarzt. 2021;92(1):1‐8. doi:10.1007/s00115-020-00920-1 32394005 PMC7808990

[34] Zernikow J , Grassow L , Gröschel J , Henrion P , Wetzel PJ , Spethmann S . Clinical application of large language models: does ChatGPT replace medical report formulation? An experience report. Inn Med (Heidelb). 2023;64(11):1058‐1064. doi:10.1007/s00108-023-01600-3 37843579

[35] Goodman RS , Patrinely JR , Stone CAJ , et al. Accuracy and reliability of chatbot responses to physician questions. JAMA Netw Open. 2023;6(10):e2336483. doi:10.1001/jamanetworkopen.2023.36483 37782499 PMC10546234

[36] Bischof T , Al Jalali V , Zeitlinger M , et al. Chat GPT vs. clinical decision support systems in the analysis of drug‐drug interactions. Clin Pharmacol Ther. 2025;117(4):1142‐1147. doi:10.1002/cpt.3585 39935064 PMC11924163

[37] Helgestad OK , Hjelholt AJ , Vestergaard SV , Azuz S , Sædder EA , Overvad TF . ChatGPT versus physician‐derived answers to drug‐related questions. Dan Med J. 2024;72(1):A05240360. doi:10.61409/A05240360 39936270

[38] Huang X , Estau D , Liu X , Yu Y , Qin J , Li Z . Evaluating the performance of ChatGPT in clinical pharmacy: a comparative study of ChatGPT and clinical pharmacists. Br J Clin Pharmacol. 2024;90(1):232‐238. doi:10.1111/bcp.15896 37626010

[39] Al‐Dujaili Z , Omari S , Pillai J , Al Faraj A . Assessing the accuracy and consistency of ChatGPT in clinical pharmacy management: a preliminary analysis with clinical pharmacy experts worldwide. Res Social Adm Pharm. 2023;19(12):1590‐1594. doi:10.1016/j.sapharm.2023.08.012 37696742

[40] Fournier A , Fallet C , Sadeghipour F , Perrottet N . Assessing the applicability and appropriateness of ChatGPT in answering clinical pharmacy questions. Ann Pharm Fr. 2024;82(3):507‐513. doi:10.1016/j.pharma.2023.11.001 37992892

[41] Roosan D , Padua P , Khan R , Khan H , Verzosa C , Wu Y . Effectiveness of ChatGPT in clinical pharmacy and the role of artificial intelligence in medication therapy management. J Am Pharm Assoc. 2024;64(2):422‐428. 10.1016/j.japh.2023.11.023 38049066

[42] Munir F , Gehres A , Wai D , Song L . Evaluation of ChatGPT as a tool for answering clinical questions in pharmacy practice. J Pharm Pract. 2024;37(6):1303‐1310. doi:10.1177/08971900241256731 38775367

[43] van Nuland M , Lobbezoo AH , van de Garde EMW , et al. Assessing accuracy of ChatGPT in response to questions from day to day pharmaceutical care in hospitals. Explor Res Clin Soc Pharm. 2024;15:100464. 39050145 10.1016/j.rcsop.2024.100464 PMC11267013

[44] van Nuland M , Erdogan A , Aςar C , et al. Performance of ChatGPT on factual knowledge questions regarding clinical pharmacy. J Clin Pharmacol. 2024;64(9):1095‐1100. doi:10.1002/jcph.2443 38623909

[45] Kataoka Y , So R . Benefits, limits, and risks of GPT‐4 as an AI Chatbot for medicine. N Engl J Med. 2023;388(25):2399. doi:10.1056/NEJMc2305286 37342939

[46] Haftenberger A , Dierks C . Legal integration of artificial intelligence into internal medicine: data protection, regulatory, reimbursement and liability questions. Inn Med (Heidelb). 2023;64(11):1044‐1050. doi:10.1007/s00108-023-01598-8 37861724

[47] Reis M , Reis F , Kunde W . Influence of believed AI involvement on the perception of digital medical advice. Nat Med. 2024;30(11):3098‐3100. doi:10.1038/s41591-024-03180-7 39054373 PMC11564086

[48] OpenAI . Introducing GPT‐5. 2025. Available at: https://openai.com/index/introducing-gpt-5/. Accessed August 14, 2025.

[49] Capoot A . OpenAI launches new GPT‐5 model for all ChatGPT users. 2025. Available at: https://www.cnbc.com/2025/08/07/openai-launches-gpt-5-model-for-all-chatgpt-users.html. Accessed August 14, 2025.

[50] Google DeepMind . Google Gemini. Available at: https://gemini.google.com/app. Accessed August 14, 2025.

[51] Meta AI . Llama 4. Available at: https://www.llama.com/. Accessed August 14, 2025.

[52] Haug CJ , Drazen JM . Artificial intelligence and machine learning in clinical medicine, 2023. N Engl J Med. 2023;388(13):1201‐1208. doi:10.1056/NEJMra2302038 36988595

[53] Krumborg JR , Mikkelsen N , Damkier P , et al. ChatGPT: first glance from a perspective of clinical pharmacology. Basic Clin Pharmacol Toxicol. 2023;133(1):3‐5. doi:10.1111/bcpt.13879 37170853

