# Supplementary material for: Human vs. artificial intelligence: Physicians outperform ChatGPT in real‐world pharmacotherapy counselling
Source: Br J Clin Pharmacol. 2025 Oct 25;92(3):891–902. doi: 10.1002/bcp.70321 (PMC12930016; doi:10.1002/bcp.70321)
Supplement: Supplementary file 1 — Data S1. Examples of errors in physician‐ and artificial intelligence chatbot‐generated responses to pharmacotherapeutic queries submitted by healthcare professionals to the clinical‐pharmacological drug information center of Hannover Medical School. Note: Queries and responses were translated from German to English with DeepL (Cologne, Germany; https://www.deepl.com/de/translator). For the selected cases, the corresponding chatbot‐ or physician‐generated responses (respectively) are juxtaposed in the last column for comparison. Please note that those responses may also contain errors. [file BCP-92-891-s001.docx]

**SUPPLEMENTARY MATERIAL 1** Examples of errors in physician- and artificial intelligence chatbot-generated responses to pharmacotherapeutic queries submitted by healthcare professionals to the clinical-pharmacological drug information center of Hannover Medical School. Note: Queries and responses were translated from German to English with DeepL (Cologne, Germany; https://www.deepl.com/de/translator). For the selected cases, the corresponding chatbot- or physician-generated responses (respectively) are juxtaposed in the last column for comparison. Please note that those responses may also contain errors.

| **Physician-generated responses** | | | | | |
| --- | --- | --- | --- | --- | --- |
| **Query** | **Response** | **Error(s)** | **Correction(s)** | **Comment** | **Corresponding chatbot-generated response** |
| Male patient, 73 years old.  Brief medical history:  Pangonarthrosis left side  Further diagnoses:  Arterial hypertension  Obesity  Post myocardial infarction  eGFR = 69 ml/min  CRP = 27.7 mg/l  Medication:  ASA 100 mg 1-0-0  Amlodipine 10 mg 1-0-1  Candesartan 16 mg 1-0-1  Moxonidine 0.3 mg 1-0-1  Torasemide 10 mg 1-0-0  Doxazosin 4 mg 1-0-1  Atorvastatin 40 mg 0-0-1  Metamizole 500 mg 2-2-2-2  Macrogol sachet 1-0-0  Hydromorphone extended release 2 mg 1-1-1  Hydromorphone 1.3 mg as required  Ropivacaine pump 4 ml/h  Tinzaparin 4500 IU 0-0-1  Question:  Interaction assessment of the drugs considering the patient’s polymedication | The following pharmacodynamic interactions must be taken into account with your patient’s current medication:  1. ASA + metamizole 🡪 decrease in the antiplatelet effect of ASA when given at the same time as metamizole. ASA should be taken at least 2 hours before metamizole.  2. Candesartan + amlodipine + moxonidine + doxazosin + torasemide 🡪 desired additive antihypertensive effects  3. Hydromorphone + ropivacaine (Naropin® pump) + metamizole 🡪 desired additive analgesic effects  Pharmacokinetic interactions are not present. | The increased risk of bleeding during treatment with ASA and tinzaparin should also be considered.  The risk of orthostatic dysregulation and possible syncope tendencies should also be taken into account when using an antihypertensive quintuple combination. | Further pharmaco­dynamic interactions (increased bleeding risk, increased risk of orthostatic dysregulation and falls) should have been addressed in the response. | Clinically, particular attention should be paid to bleeding complications and possible sedation under long-term opioid therapy in advanced age with regard to possible drug interactions. | Here are potential interactions that should be considered:  1. ASA (acetylsalicylic acid) may increase the risk of bleeding, especially when taken with anticoagulants such as tinzaparin. It is important to monitor the risk of bleeding carefully.  2. Amlodipine, candesartan, and moxonidine are used to treat high blood pressure. There are usually no serious interactions between these medications. However, blood pressure, kidney function, and electrolyte levels should be monitored.  3. Torasemide is a diuretic used to treat fluid retention. Concomitant use of torasemide and doxazosin (alpha-blocker) may lead to an increased drop in blood pressure. This should be monitored, especially when taking the first doses or when adjusting the dose.  4. Atorvastatin is used to lower cholesterol levels. There are no known serious interactions with the other medications listed. However, atorvastatin can affect muscle function and cause muscle pain (myalgia).  5. Metamizole is a painkiller and may increase the risk of bleeding, especially when combined with anticoagulants such as tinzaparin. The combination should be used with caution and the risk of bleeding should be monitored. |
| A 61-year-old male patient suffers from F33.1.  His blood pressure is uncontrollable despite his blood pressure medication being quite extensive, and he is still hypertensive on desvenlafaxine. As he complains of a pronounced lack of drive, I would like to start him on an activating medication. In my opinion, the SSRI with the strongest drive is escitalopram (also in conjunction with sertraline), so I am endeavouring to switch to this drug.  His current medication is:  Lithium, lercanidipine, desvenlafaxine, moxonidine, bisoprolol, candesartan, L-tyhroxine, Ezehron duo  We have already discontinued trimipramine and allopurinol and are currently phasing out desvenlafaxine. | Escitalopram is to be assessed more favourably than desvenlafaxine with regard to the patient’s arterial hypertension due to the lack of a noradrenergic component. While high blood pressure is not listed as an ADR in the SmPC of escitalopram, a separate section is dedicated to blood pressure in the chapter “Special warnings and precautions for use” in the SmPC of desvenlafaxine. Increased blood pressure occurs frequently (i.e., in 1 to 10 % of treated patients) during therapy with desvenlafaxine.  The fact that the patient’s blood pressure is not adequately controlled despite antihypertensive quadruple therapy (lercanidipine, candesartan, bisoprolol, moxonidine) raises the question of whether secondary causes of arterial hypertension such as renal artery stenosis etc. have already been ruled out? If not, this would certainly be the domain of the patient’s general practitioner/internist and not the task of psychiatry.  However, you can also initially observe the development of blood pressure after discontinuation of desvenlafaxine; discontinuation of desvenlafaxine alone may already be sufficient to improve the patient’s blood pressure situation.  Generally speaking, bisoprolol and moxonidine are not the drugs of first choice in hypertension therapy. However, it is questionable whether a thiazide diuretic would be the better alternative in this specific case in view of the lithium medication. If a decision is made in favor of a switch, it should be noted that bisoprolol should be gradually discontinued a few days before moxonidine (not vice versa), as otherwise there is a risk of rebound hypertension.  With regard to QTc interval prolongation, there is no contraindication for escitalopram in view of the concomitant medication reported. Irrespective of this, the usual precautionary measures such as monitoring the QTc interval before and during therapy with escitalopram apply. | Under long-term treatment with moxonidine, depressive moods are a possible ADR, which is why this reserve drug for hypertension treatment should be discussed particularly critically here.  Reference should also be made to the direct healthcare professional communication (DHPC), which considers a combination of escitalopram with other QTc-prolonging drugs to be contraindicated. | Depression as a possible ADR of moxonidine as well as the DHPC for escitalopram (referring to the risk of QTc interval prolongation) could have been mentioned in the response. | In this case, a re-evaluation of the antihypertensive medication with the help of a cardiologist appears particularly useful. The indication and response to the medication with lithium should also be checked, as it is only authorized for augmentation treatment in severe depression; in the current case, however, moderate depression is described (F33.1). | Escitalopram is a selective serotonin reuptake inhibitor (SSRI) and may have a stimulating effect in some patients. It is often prescribed for depression to improve motivation. However, it is important to consider possible interactions with other medications.  One potential interaction between escitalopram and certain medications is a prolongation of the QT interval on the electrocardiogram (ECG). A prolonged QT interval can lead to heart rhythm disturbances. It is important to consider this in patients who are already prone to heart problems or are taking other medications that can prolong the QT interval.  Since your patient is already taking several medications, including desvenlafaxine, potential interactions with escitalopram should be carefully monitored.  It is important that your patient undergoes regular check-ups and follow-ups with a physician to monitor their blood pressure and depressive symptoms. Only a physician can make an accurate assessment and recommend appropriate treatment tailored to the individual needs of the patient. |
| 78-year-old female patient in trauma surgery  eGFR = 61 ml/min  CRP = 115.3 mg/l  Brief medical history:  Fall in nursing home, known paresis right side  Diagnosis:  Hip fracture, pneumonectomy right side  Medication:  Salbutamol inhalation 3x/d  O_2_ 3 l/min  Nebivolol 5 mg 1-0-0  Ramipril 2.5 mg 1-0-0  Atorvastatin 20 mg 0-0-1  Novalgin 40 gtt. 1-1-1-1  Pantoprazole 20 mg 1-0-0  Movicol sachet 1-0-0  Kalinor extended release 600 mg 0-1-0  Spiolto Respimat 2.5 µg/2.5 µg 1-0-1  Corneregel eye drops 1-1-1  Hydromorphone extended release 2 mg 1-1-1  Hydromorphone 1.3 mg up to 6x/d  Innohep 4500 IU 0-0-1  Question:  Polypharmacy counselling with potential interactions | Under the current medication of your patient (78 years, eGFR = 61 ml/min), a reduction in the effect of olodaterol (in Spiolto Respimat) due to the administration of a beta-blocker (here: cardioselective beta-blocker nebivolol) cannot be ruled out.  Metamizole should only be administered to geriatric patients in the current high dosage (4 g/d) for a short period. | Attention should also be paid to the interaction of Kalinor (potassium) with ramipril and the associated risk of hyperkalemia. | The potential interaction between Kalinor and ramipril should have also been addressed in the response. | Regular determination of electrolyte levels is essential in this case to prevent hyperkalemia.  In addition, the analgesic therapy consisting of metamizole and hydromorphone should be re-evaluated and de-escalated during the course of treatment, especially because of the risk of excessive sedation with opioids in geriatric patients. | You are seeking advice on polypharmacy and potential interactions in a 78-year-old female patient with various medical conditions and medications. I will attempt to identify some potential interactions and offer possible considerations.  Some possible interactions and considerations may include:  1. Nebivolol and salbutamol: Both medications can affect blood pressure. Blood pressure monitoring may be necessary, as interactions between beta-blockers such as nebivolol and bronchodilator medications such as salbutamol may occur.  2. Ramipril and hydromorphone: Ramipril is an ACE inhibitor and hydromorphone is an opioid analgesic. Both can affect blood pressure and there is potential for interactions. Careful monitoring of blood pressure and kidney function is important.  3. Atorvastatin and Innohep: Innohep is a low molecular weight heparin that prevents blood clotting. There may be potential interactions with statins such as atorvastatin. This could increase the risk of bleeding. Coagulation function should be monitored.  4. Pantoprazole and Novalgin: Pantoprazole is a proton pump inhibitor that reduces stomach acid production. Novalgin is a painkiller. Pantoprazole may increase the effect of Novalgin, which could lead to an increased risk of bleeding.  5. Kalinor extended release and Spiolto Respimat: Spiolto Respimat contains tiotropium and olodaterol for the treatment of COPD. The simultaneous use of potassium preparations such as Kalinor and potassium-sparing diuretics with tiotropium can lead to increased potassium levels.  6. Movicol and hydromorphone: Movicol is a laxative. It can affect the effect of hydromorphone by influencing its absorption in the gastrointestinal tract. The effectiveness of hydromorphone could be impaired. |
| **Artificial intelligence chatbot-generated responses** | | | | | |
| **Query** | **Response** | **Error(s)** | **Correction(s)** | **Comment** | **Corresponding physician-generated response** |
| 85-year-old female patient  eGFR = 85 ml/min  Cervical collar; indwelling catheter; fixator for the pelvis  Diagnosis:  Secondary dislocated fracture of the left proximal phalanx D1 after secondary conversion of K-wires to a plate osteosynthesis  Medication:  NaCl 0.9 % inhalation 3x/d  Bisoprolol 2.5 mg 1-0-0  Atorvastatin 40 mg 0-0-1  L-thyroxine 50 µg 1-0-0  Novalgin 500 mg 2-2-2-2  Godamed 100 mg 1-0-0  Pantoprazole 40 mg 1-0-0  Movicol sachet 1-0-0  Palladon extended release 2 mg 1-1-1  Palladon 1.3 mg as required  Tazobac 4.5 g i.v. 1-1-1  Innohep 4500 IU 0-0-1  Question:  Polymedication – interaction counselling | When analyzing the 85-year-old patient’s polymedication, several medications are found. Here is a breakdown of the individual medications and possible interactions:  1. NaCl 0.9 % inhalation: this medication is an isotonic saline solution and generally has no known interactions with other medications.  2. Bisoprolol 2.5 mg: Bisoprolol is a beta-blocker and is often used to treat high blood pressure and heart disease. Concomitant use of bisoprolol and novalgin (metamizole) may result in an increased antihypertensive effect. Regular monitoring of blood pressure is important to avoid excessive lowering of blood pressure.  3. Atorvastatin 40 mg: Atorvastatin is a cholesterol-lowering agent and is used to treat high cholesterol levels. There are no known interactions between atorvastatin and the other medicines listed.  4. L-thyroxine 50 µg: L-thyroxine is a thyroid hormone and is used to treat hypothyroidism. There are no known interactions between L-thyroxine and the other medicines listed.  5. Novalgin 500 mg: Novalgin is a painkiller (metamizole) and has a potential interaction with other medications. As already mentioned, a combination of Novalgin with bisoprolol can lead to an increased antihypertensive effect. It is important to monitor blood pressure regularly and adjust the dosage accordingly.  6. Godamed 100 mg: Godamed (diclofenac) is a non-steroidal anti-inflammatory drug (NSAID). If Godamed and Tazobac (an antibiotic) are used at the same time, there is an increased risk of gastrointestinal bleeding. The combination should be avoided or closely monitored.  7. Pantoprazole 40 mg: Pantoprazole is a proton pump inhibitor used to treat gastrointestinal ulcers and reflux disease. There are no known interactions between pantoprazole and the other medicines listed.  8. Movicol sachet: Movicol is a medicine used to treat constipation. There are no known interactions between Movicol and the other medicines listed.  9. Palladon extended release 2 mg: Palladon (hydromorphone) is a strong painkiller (opioid). It should be used with caution, especially in elderly patients, as it can have a sedative effect and can lead to respiratory depression. The concomitant use of Palladon with other sedative drugs such as Tazobac may increase these effects. Regular monitoring of breathing and sedation status is important.  10. Palladone 1.3 mg as needed: This medicine contains the same active substance (hydromorphone) as palladone extended release 2 mg and should be used according to the same precautions.  11. Tazobac 4.5 g i.v.: Tazobac is an antibiotic (tazobactam/pipera­cillin) and is used to treat infections. There are no known interactions between Tazobac and the other medicines listed.  12. Innohep 4500 IU: Innohep (tinzaparin) is a low molecular weight heparin and is used to prevent and treat blood clots. There are no known interactions between Innohep and the other medications listed.  It is important to note that this is a general assessment and other individual factors must be taken into account. | Interaction of bisoprolol with metamizole is incorrect.  Interaction proton pump inhibitor + L-thyroxine not recognized.  Clinically important interaction acetylsalicylic acid (Godamed) + metamizole was not addressed.  Godamed 100 mg (acetylsalicylic acid) is labelled as diclofenac.  No potential interactions of pantoprazole mentioned.  Tazobac labelled as a sedative drug.  Incorrect interaction diclofenac + tazobac: increased risk of gastrointestinal bleeding. | There is no known clinically relevant interaction of bisoprolol with metamizole.  Proton pump inhibitors can reduce the absorption of L-thyroxine.  Clinically important interaction between acetylsalicylic acid and metamizole: If taken at the same time, the therapeutically desired effect of acetylsalicylic acid may be diminished.  Godamed is the trade name for acetylsalicylic acid.  Tazobac is not a sedative agent.  The risk of bleeding associated with the administration of Innohep was not mentioned. | The response contradicts itself. On the one hand, interactions of Tazobac (sedation and bleeding risk) are posited, while in the recommen­dation pertaining to Tazobac itself it is stated: “no known interactions”. | The current therapy for your patient (85 years old, eGFR = 85 ml/min) has the following interactions:  1. L-thyroxine + pantoprazole 🡪 Simultaneous administration can lead to reduced plasma concentrations of L-thyroxine. For this reason, the two drugs should be taken as far apart as possible.  2. ASA + metamizole 🡪 Simultaneous administration of metamizole may abolish the antiplatelet effect of ASA. It is recommended that the two drugs be taken at different times (ASA at least two hours before or after metamizole). |
| 88-year-old male patient in the trauma surgery department  eGFR: unknown  CRP: unknown  Brief history: fall during anticoagulation with head impact  Diagnosis: Dens fracture type 2b, Gehweiler 3 fracture  Medication:  Metformin 500 mg 1-0-1, currently paused  ASA 100 mg 1-0-0  Candesartan 8 mg 1-0-0  Donepezil 5 mg 0-0-1  Levodopa/benserazide 50/14.27 mg 1-1-1  Magnesium 400 mg 0-0-1  Mirtazapine 15 mg 0-0-1  Pipamperone 40 mg 0-0-1  Simvastatin 30 mg 1-0-0  Sitagliptin 50 mg 1-0-0  Vitamin D_3_ 4000 IU 0-0-1  Macrogol sachet 1-0-0  Momegalen ointment (mometasone)  Levodopa/benserazide 100/25 mg 0-0-1  Laxans 0-0-0-14 gtt.  Palladon 2 mg 1-0-1  Palladon 1.3 mg up to 4x/d  Actrapid 7-12-17 IU  Lantus 0-0-10 IU  Nutriflex peri 40 ml/h  Tazobac 4.5 g 1-1-1  Dulcolax suppository if required  Pipamperone 5 ml 3x/d, 4 h apart  Question: Polypharmacy, interaction counselling | There are a number of medications in this 88-year-old patient’s medication list that should potentially be reviewed due to polypharmacy and possible drug interactions. Here are some considerations:  1. Metformin: It has been paused, which may be appropriate given the current health status and likelihood of contrast media administration.  2. ASA (acetylsalicylic acid): This drug should be used with caution due to the increased risk of bleeding in elderly patients. It may interact with other medications, particularly blood thinners such as Tazobac (probably tazobactam/pipera­cillin) and Lantus (insulin glargine).  3. Candesartan: This blood pressure medication should be monitored in elderly patients to avoid low blood pressure, especially in combination with levodopa.  4. Donepezil: This medication is used to treat dementia and has the potential to interact with other medications on the list. The patient’s cognitive function should be monitored.  5. Levodopa/bense­razide: The combination of levodopa/benserazide can cause side effects such as dizziness, especially in combination with other drugs that affect blood pressure, such as candesartan. Dose adjustment and monitoring for movement disorders is also important.  6. Simvastatin: This drug can cause muscle problems and should be used with caution, especially in elderly patients.  7. Sitagliptin: The use of sitagliptin should be based on renal function, which is not known here. It should be checked with metformin.  8. Palladone (hydromorphone): This potent analgesic should be carefully dosed and monitored, especially in combination with other analgesics such as Actrapid.  9. Macrogol: A laxative should be used when needed and dosing should be monitored appropriately.  10. Tazobac (tazobactam/pipera­cillin): This antibiotic should be used for the treatment of infections and should be carefully monitored, especially if the patient is taking other medications.  11. Mirtazapine and pipamperone: these two drugs can have a sedative effect and should be checked for interactions with other sedative drugs such as palladone and levodopa/bensera­zide.  12. Vitamin D_3_: Vitamin D supplementation should be adjusted to renal function, which is not known in this case.  13. Dulcolax suppository: This laxative should be used when necessary, but the dosage should be carefully monitored.  It is extremely important that this patient is cared for by an experienced doctor who can take into account the overall situation and the interactions between these medications. Regular monitoring, especially of kidney function and blood pressure, is crucial. Medication adjustments and dose adjustments should be considered to ensure the safety and efficacy of the therapy. | Tazobac is described as a blood thinner.  The terminology “blood thinner” is wrong.  Lantus (insulin glargine) is not a blood thinner.  Actrapid (human insulin) is not an analgesic. | Tazobac (pipera­cillin/tazobac­tam) is an antibiotic.  Lantus is a long-acting insulin analogue.  Actrapid is a short-acting human insulin. | It is striking in this response that three drug brand names could not be assigned correctly to their corresponding generic names, which negatively impacts on pharmaco­therapy safety as drugs might be confounded by the attending healthcare professionals.  The term “blood thinner” should not be used as it is a colloquial expression and pharmaco­dynamically wrong; the term anticoagulant should be preferred in interprofessional communication.  Besides, it should have been recommended taking simvastatin in the evening as the drug is more effective then.  Pipamperone may trigger a cogwheel phenomenon in a patient with Parkinson’s disease (the presence of which can be indirectly inferred from the patient’s list of medication containing levodopa/bense­razide)—this has not been addressed in the response. | The medication of your patient (88 years old) shows the following pharmacodynamic interactions:  1. Under combined therapy with hydromorphone, mirtazapine and pipamperone, an increase in CNS depressant effects is to be expected.  2. Pipamperone can cause rigidity, akathisia or dyskinesia and accordingly may weaken the effect of levodopa.  3. The risk of orthostatic hypotension must be taken into account during therapy with candesartan and levodopa. |
| 57-year-old male patient undergoing cardiothoracic and vascular surgery  eGFR = 9 ml/min  Brief medical history: Massive reaction to Marcumar. Frequent derailment with nosebleeds. Marcumar necessary for 3 months in case of biological mitral valve replacement and tricuspid valve reconstruction status post mitral valve replacement + tricuspid valve reconstruction+ rethoracotomy for hematothorax  ASA 100 mg 1-0-0  Magnesium verla 2-2-2  Prednisolone 5 mg 3-0-0  Fosrenol 1000 mg 1-1-1  L-thyroxine 25 μg 1-0-0  Nebivolol 5 mg 1/2-0-0  Roxadustat 100 mg for dialysis  Temgesic 0.2 mg as needed  Flucloxacillin 1 g 1-0-1  Meronem 1 g 1-0-1  I would like to receive an opinion and therapy recommendation for the patient’s frequent INR derailment after only 1/4 tablet of Marcumar. Thank you very much. | It appears that the patient has a complex medical history and suffers from various conditions that require careful drug treatment. The frequent INR derailment despite a low dose of Marcumar (1/4 tablet) is worrying and requires a review and adjustment of therapy.  It is important that the patient is under the care of an experienced physician, preferably a hemostaseologist or cardiologist who specializes in blood coagulation. Here are some considerations and recommendations for further treatment:  1. Review causes of INR derailment: The physician should review the possible causes of INR derailment, including possible interactions with other medications, dietary factors, or other underlying health conditions.  2. Alternative anticoagulants: For patients who do not respond well to warfarin (Marcumar), alternative oral anticoagulants such as dabigatran, rivaroxaban or apixaban may be considered. These drugs have a predictable effect and do not usually require frequent INR monitoring.  3. Regular monitoring and dose adjustment: Even if alternative anticoagulants are used, regular monitoring of blood coagulation levels is important. Dose adjustment should be based on this monitoring to achieve a therapeutic range (INR target) and prevent bleeding or thromboembolic events.  4. Check interactions with other medications: It is important to ensure that there are no interactions between Marcumar and other medications that the patient is taking. In particular, attention should be paid to interactions with current medications such as prednisolone, nebivolol, roxadustat and others.  5. Diet and lifestyle: The patient should be instructed to maintain a constant vitamin K intake, as fluctuations in vitamin K intake can influence the effect of warfarin. Alcoholic beverages should also be consumed in moderation, as alcohol can influence the coagulation factor.  6. Close monitoring: it is important that the patient is regularly monitored by a healthcare provider to keep blood clotting levels within the target range and avoid potential complications. | INR monitoring is recommended also for alternative anticoagulants such as dabigatran, rivaroxaban or apixaban.  Marcumar is used as a trade name for warfarin. | INR monitoring is not indicated for direct thrombin inhibitors or direct factor Xa inhibitors.  Marcumar is the trade name of phenpro­coumon, not warfarin. | The indication for the combined use of phenprocoumon and low-dose ASA is unclear. This should have been recognized and should have prompted a question addressed back to the inquiring healthcare professional.  It is unknown whether the patient suffers from atrial fibrillation or has a history of thromboembolic events, which would be an indication for anticoagulation. As the mitral valve is biological and not mechanical, anticoagulation would not be indicated. If atrial fibrillation were present, for example, a switch to a DOAC (off-label due to stage-5 renal insufficiency) should be considered. Importantly, INR monitoring is neither necessary nor useful for DOACs. The chatbot suggests a wrong monitoring technique here, which may lead the attending healthcare professionals to draw false therapeutic conclusions. | Meropenem can increase the anticoagulant effect of vitamin K antagonists (in this case phenprocoumon). The risk depends on the age and condition of the patient as well as the underlying infection. Furthermore, there is a risk of thrombocytopenia or epistaxis, especially in patients with impaired renal function (in this case: eGFR = 9 ml/min).  Due to the severely impaired renal function, we recommend reducing the dose of meropenem to 1 g every 24 hours.  To stabilize bleeding during therapy with phenprocoumon and ASA, we recommend consulting the expertise of the hemostasis clinic. |

Abbreviations: ACE, angiotensin-converting enzyme; ADR, adverse drug reaction; ASA, acetylsalicylic acid; CNS, central nervous system; COPD, chronic obstructive pulmonary disease; CRP, C-reactive protein; DHPC, direct healthcare professional communication; DOAC, direct oral anticoagulant; ECG, electrocardiogram; eGFR, estimated glomerular filtration rate; F33.1, ICD-10 (International Statistical Classification of Diseases and Related Health Problems 10th Revision) code for recurrent depressive disorder, current episode moderate; gtt. (Latin: *guttae*), drops; INR, international normalized ratio; IU, international units; NSAID, non-steroidal anti-inflammatory drug; SmPC, summary of product characteristics; SSRI, selective serotonin reuptake inhibitor
